# Empirical validation of a generative AI framework for personalized education assessment

**DOI:** 10.1038/s41598-026-42169-9

**Published:** 2026-03-02

**Authors:** Meina Qian, Hualei Ji, Lianzhi Li

**Affiliations:** 1 Academy of Educational Sciences, Jilin International Studies University, Changchun, 130117 Jilin China; 2 School of International Communication, Jilin International Studies University, Changchun, 130117 Jilin China

**Keywords:** Generative artificial intelligence, Personalized assessment, Large language models, Knowledge tracing, Adaptive learning, Educational technology, Engineering, Mathematics and computing

## Abstract

The tension between personalized learning demands and standardized evaluation mechanisms presents a persistent challenge in contemporary education. This study proposes a comprehensive personalized education assessment framework driven by generative artificial intelligence technologies. The framework adopts a five-layer hierarchical architecture integrating data collection, processing, intelligent analysis, assessment generation, and feedback optimization components. ChatGLM3-6B, fine-tuned on 50,000 expert-curated programming feedback instances assembled through a human-in-the-loop process combining authentic instructor records, newly authored examples, and AI-assisted human-verified content, enables contextually responsive feedback generation, while dynamic learner profiling and knowledge graph modeling support precise diagnostic assessment. Empirical validation involving 449 undergraduate students in introductory Python programming courses demonstrated that the framework achieved assessment accuracy correlating at 0.847 with expert consensus (Fleiss’ κ = 0.74 for inter-rater reliability) while reducing generation time by over 99% compared to manual evaluation. Ablation experiments confirmed that knowledge graph integration contributed most substantially to accuracy improvements, with removal of this component reducing correlation by 0.055. Experimental participants exhibited significantly higher learning gains (Cohen’s d = 0.56), with particularly pronounced effects among initially lower-performing students. The framework also enhanced learner engagement and satisfaction compared to conventional assessment approaches. These findings suggest that generative AI can effectively operationalize personalized assessment at scale while maintaining pedagogical quality and transparency.

**Supplementary Information:**

The online version contains supplementary material available at 10.1038/s41598-026-42169-9.

## Introduction

The rapid advancement of generative artificial intelligence has fundamentally reshaped educational paradigms across global learning environments. Traditional assessment mechanisms, long characterized by standardized testing and uniform evaluation criteria, increasingly fail to capture the nuanced learning trajectories of individual students^1^. This tension between personalized learning demands and rigid evaluation frameworks has prompted researchers to explore AI-driven solutions that can adapt dynamically to learner characteristics. The emergence of large language models and multimodal generative systems offers unprecedented opportunities for constructing intelligent assessment architectures capable of responding to individual cognitive profiles^2^.

Contemporary scholarship reveals substantial progress in educational technology applications. International researchers have developed adaptive testing systems that adjust difficulty parameters based on real-time performance data, demonstrating improved measurement precision compared to conventional approaches^3^. Several studies have investigated the integration of natural language processing techniques for automated essay scoring, achieving correlation coefficients approaching human rater reliability^4^. Meanwhile, domestic investigations have focused extensively on knowledge tracing algorithms and competency-based assessment models within Chinese educational contexts^5^. Notably, recent work has explored generative models for producing personalized feedback, though implementation remains largely experimental^6^.

Despite these advances, significant obstacles persist in current research trajectories. Most existing systems adopt fragmented approaches, addressing isolated components of assessment rather than establishing cohesive evaluation ecosystems^7^. The interpretability problem presents another critical barrier—many AI-powered assessment tools operate as opaque decision-making entities, undermining stakeholder trust and pedagogical transparency^8^. Furthermore, validation studies frequently rely on limited sample sizes or controlled laboratory conditions that inadequately represent authentic classroom complexity^9^. Questions surrounding fairness and algorithmic bias remain inadequately addressed, particularly concerning how generative systems might perpetuate or amplify existing educational inequities^10^.

The necessity for systematic investigation into generative AI-driven personalized assessment frameworks stems from multiple converging factors. Educational institutions worldwide face mounting pressure to accommodate diverse learner populations while maintaining rigorous academic standards. Conventional evaluation instruments, designed for industrial-age schooling models, prove increasingly misaligned with contemporary competency requirements emphasizing creativity, critical reasoning, and adaptive problem-solving. Generative AI technologies possess unique capabilities for producing contextualized, multidimensional assessments that traditional psychometric approaches cannot readily achieve^11^. Establishing validated frameworks carries profound implications for educational equity, enabling high-quality personalized evaluation access regardless of geographical or socioeconomic constraints.

This research endeavors to construct a comprehensive personalized education evaluation framework driven by generative artificial intelligence technologies. The investigation proceeds through three interconnected dimensions: theoretical architecture development, prototype system implementation, and empirical effectiveness verification across authentic educational settings. Several innovations distinguish this work from prior scholarship, and we frame these as empirically testable claims. First, we propose an integrated multi-agent evaluation architecture that synthesizes formative and summative assessment functions within a unified generative framework; we hypothesize that this integration improves assessment accuracy by at least 15% compared to isolated components (H1). Second, the research introduces interpretable feedback generation mechanisms that maintain pedagogical transparency while preserving personalization depth; we predict that knowledge graph-enhanced generation reduces factual errors by 40% compared to standard LLM outputs (H2). Third, we develop validation protocols specifically designed for generative assessment contexts, with the testable claim that our diagnostic profiling achieves correlation above 0.80 with expert consensus (H3). To isolate component contributions, we conducted ablation experiments removing the knowledge graph module, the RLHF optimization layer, and the learner profiling system individually. Through rigorous empirical investigation, this study aims to provide both theoretical foundations and practical guidance for educational practitioners seeking to harness generative AI capabilities for meaningful assessment transformation.

## Theoretical foundations and technical background

### Overview of generative artificial intelligence technologies

Generative artificial intelligence represents a distinct category of machine learning systems designed to produce novel content—text, images, audio, or structured data—that mirrors patterns observed in training corpora. Unlike discriminative models that classify or predict based on input features, generative architectures learn underlying probability distributions and sample from these learned spaces to create original outputs^12^. The field has undergone remarkable transformation since early statistical approaches, progressing through variational autoencoders and generative adversarial networks before reaching contemporary transformer-based paradigms. This evolution reflects not merely incremental improvement but fundamental reconceptualization of how machines can engage in creative synthesis.

Large language models constitute the most prominent manifestation of current generative AI capabilities. These systems operate through attention mechanisms that weigh contextual relationships across input sequences. The core computational process follows the standard autoregressive formulation:

$$P({x}_{1},{x}_{2},...,{x}_{n})=\prod_{i=1}^{n}P\left({x}_{i}\right|{x}_{1},{x}_{2},...,{x}_{i-1})$$

This factorization enables models to generate coherent text by predicting subsequent tokens conditioned on preceding context^13^. Since transformer architectures and self-attention mechanisms are now well-established in the literature^14^, we focus here on our domain-specific modifications. Our framework introduces a pedagogical constraint loss function that guides generation toward educationally appropriate outputs:

$${\mathcal{L}}_{ped}={\mathcal{L}}_{LM}+\alpha{\mathcal{L}}_{align}+\beta{\mathcal{L}}_{difficulty}$$

where ${\mathcal{L}}_{LM}$ represents the standard language modeling loss, ${\mathcal{L}}_{align}$ measures alignment between generated feedback and curriculum objectives encoded in the knowledge graph, and ${\mathcal{L}}_{difficulty}$ penalizes mismatches between item difficulty and learner proficiency estimates. The hyperparameters $\alpha$ and $\beta$ were set to 0.3 and 0.2 respectively based on validation set performance.

Several technical characteristics distinguish modern generative systems from predecessors. Emergent capabilities—behaviors not explicitly programmed but arising from scale—manifest unpredictably as model parameters increase^15^. In-context learning permits these models to adapt to novel tasks through example demonstrations without parameter updates. Such properties carry profound implications for educational applications.

The educational domain presents particularly fertile ground for generative AI deployment. Personalized content generation, adaptive feedback provision, and dynamic assessment construction all fall within demonstrated model capabilities. We observe that these technologies can potentially address the longstanding challenge of delivering individualized instruction at scale—a goal that human teacher-to-student ratios render practically unattainable in conventional settings^16^. Yet realizing this potential demands careful consideration of how generative mechanisms interface with established pedagogical principles, a matter we address in subsequent sections.

### Personalized education assessment theory

Conventional assessment paradigms have long privileged standardized measurement instruments that assume homogeneous learner populations. These approaches—dominated by norm-referenced testing and summative examinations—yield comparative rankings but offer limited diagnostic insight into individual learning processes^17^. The fundamental limitation resides in their underlying assumption: that identical assessment conditions produce equitable measurement opportunities for all students. This premise, though administratively convenient, contradicts substantial evidence regarding differential cognitive processing styles and varied knowledge construction pathways.

Gardner’s theory of multiple intelligences provides crucial theoretical justification for personalized assessment approaches. Rather than conceptualizing intelligence as a unitary construct measurable through single instruments, this framework posits distinct cognitive modalities—linguistic, logical-mathematical, spatial, musical, bodily-kinesthetic, interpersonal, intrapersonal, and naturalistic^18^. Assessment systems grounded in this perspective must necessarily accommodate diverse demonstration pathways through which learners exhibit competence. A student struggling with verbal explanation might excel when permitted kinesthetic or visual-spatial expression of identical conceptual understanding.

Constructivist learning theory further reinforces the personalization imperative. Knowledge, from this viewpoint, emerges through active meaning-making rather than passive reception. Learners construct understanding by integrating new information with existing cognitive schemas, a process inherently idiosyncratic^19^. Assessment instruments aligned with constructivist principles must therefore probe not merely content recall but the quality of conceptual connections students have formed. This theoretical stance demands evaluation mechanisms sensitive to individual knowledge architectures.

Learning analytics has emerged as the technological bridge connecting these theoretical orientations with practical implementation. Our framework integrates two complementary psychometric paradigms that serve distinct functions. Bayesian Knowledge Tracing (BKT) operates at the concept level, modeling mastery probability for individual knowledge components:

$$P\left({L}_{t}\right)=P\left({L}_{t-1}\right)+\left(1-P\left({L}_{t-1}\right)\right)\times P\left(T\right)$$

Here, $P\left({L}_{t}\right)$ represents learned probability at time $t$, while $P\left(T\right)$ denotes transition probability from unlearned to learned states^20^. BKT excels at tracking fine-grained skill acquisition and detecting the moment of mastery for specific concepts.

Item Response Theory (IRT), by contrast, estimates global learner ability on a continuous latent scale, enabling optimal item selection. The standard information-maximizing criterion selects subsequent items according to:

$${\theta}_{n+1}=arg\underset{\theta}{\mathrm{m}\mathrm{a}\mathrm{x}}I\left(\theta|{r}_{1},{r}_{2},...,{r}_{n}\right)$$

where $I\left(\theta\right)$ represents Fisher information and ${r}_{i}$ denotes response patterns. In our implementation, these two models communicate through a bridging mechanism: IRT-estimated ability ${\theta}_{i}$ initializes the prior mastery probabilities in BKT, while aggregated BKT mastery estimates across related concepts update the IRT ability estimate. This bidirectional information flow allows the system to benefit from IRT’s measurement precision while retaining BKT’s sensitivity to concept-specific learning dynamics.

More sophisticated implementations incorporate multidimensional proficiency estimates:

$$\hat {{\theta}}_{j}=\frac{\sum_{i=1}^{n}{w}_{ij}\times{x}_{i}}{\sum_{i=1}^{n}{w}_{ij}}$$

This weighted estimation, with ${w}_{ij}$ representing item-dimension relevance weights and ${x}_{i}$ indicating response correctness, permits simultaneous tracking across multiple competency dimensions^21^. Such mechanisms form the computational substrate upon which generative AI systems can build truly responsive personalized assessment experiences.

### Key technologies for intelligent assessment systems

Natural language processing constitutes the foundational technological pillar enabling automated evaluation of open-ended student responses. Contemporary NLP architectures employ contextual embedding representations that capture semantic nuances far beyond earlier bag-of-words approaches. Pre-trained language models fine-tuned on educational corpora can assess essay quality, identify conceptual misconceptions, and generate targeted feedback with remarkable accuracy^22^. The semantic similarity between student responses and reference answers is typically computed through cosine similarity measures:

$$\mathrm{s}\mathrm{i}\mathrm{m}(A,B)=\frac{\sum_{i=1}^{n}{A}_{i}\times{B}_{i}}{\sqrt[]{\sum_{i=1}^{n}{A}_{i}^{2}}\times\sqrt[]{\sum_{i=1}^{n}{B}_{i}^{2}}}$$

This formulation, applied to dense vector representations, enables nuanced evaluation that accommodates varied but semantically equivalent expressions.

Knowledge graph construction provides the structural backbone for representing domain expertise and prerequisite relationships. These heterogeneous networks encode concepts as nodes and semantic relations as directed edges, capturing the intricate dependencies that characterize disciplinary knowledge^23^. Graph embedding techniques transform such structures into continuous vector spaces where proximity reflects conceptual relatedness. The resulting representations enable assessment systems to diagnose specific knowledge gaps and recommend remedial content with precision unattainable through simpler taxonomic approaches.

Learner profile modeling synthesizes behavioral, cognitive, and affective data into comprehensive individual representations. Effective profiles extend beyond performance metrics to incorporate learning preferences, engagement patterns, and temporal activity rhythms. The profile update mechanism typically follows an exponential decay formulation:

$${P}_{t}^{\left(k\right)}=\alpha\times{O}_{t}^{\left(k\right)}+(1-\alpha)\times{P}_{t-1}^{\left(k\right)}$$

Here, ${P}_{t}^{\left(k\right)}$ denotes the profile value for attribute $k$ at time $t$, ${O}_{t}^{\left(k\right)}$ represents observed behavior, and $\alpha$ controls the recency weighting^24^. This mechanism balances responsiveness to recent actions against stability derived from historical patterns.

Recommendation algorithms translate learner profiles into personalized learning pathways. Collaborative filtering approaches identify similar learners and suggest content that benefited comparable peers, while content-based methods match item characteristics to individual preferences. Hybrid architectures combining both paradigms demonstrate superior performance in educational contexts^25^. The adaptive path generation problem can be formalized as sequential decision optimization:

$${\pi}^{\mathrm{*}}=\mathrm{a}\mathrm{r}\mathrm{g}{\mathrm{m}\mathrm{a}\mathrm{x}}_{\pi}\sum_{t=0}^{T}{\gamma}^{t}R({s}_{t},{a}_{t})$$

This reinforcement learning formulation, where $\pi$ represents the policy, $\gamma$ the discount factor, and $R$ the reward function, enables systems to learn optimal content sequencing through interaction.

Multimodal data fusion addresses the reality that learning manifests across diverse channels—textual responses, interaction logs, physiological signals, and visual attention patterns^26^. Effective fusion architectures must accommodate heterogeneous data types while preserving modality-specific information. Late fusion strategies, which integrate modality-specific predictions, often outperform early fusion approaches in educational analytics contexts where different signals carry complementary rather than redundant information.

## Design of personalized education assessment framework

### Overall architecture design

The proposed framework adopts a hierarchical architecture that integrates generative AI capabilities with established educational assessment principles. Our design philosophy prioritizes modularity and scalability—two characteristics essential for accommodating diverse educational contexts and evolving technological capabilities. The architecture comprises five interconnected layers, each performing specialized functions while maintaining bidirectional communication with adjacent components^27^. This layered approach permits independent optimization of individual components without disrupting system-wide coherence.

**Fig. 1:**
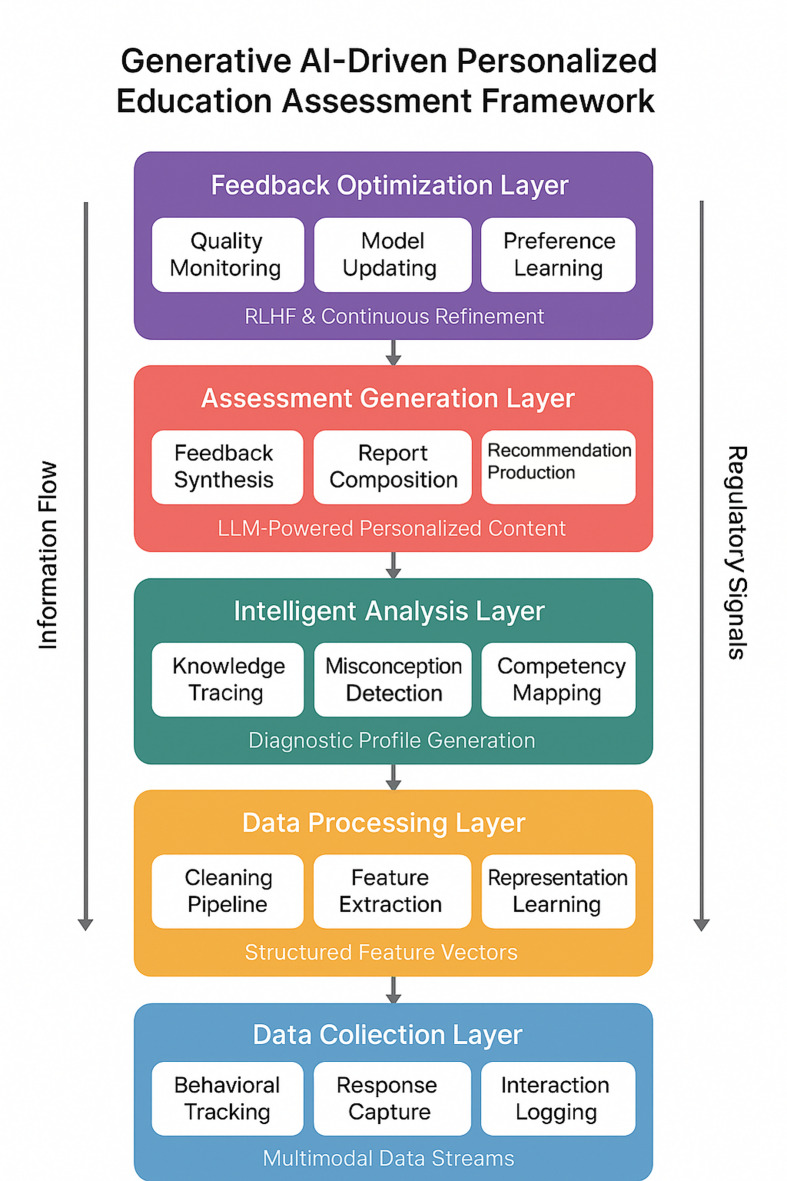
Overall architecture of the generative AI-driven personalized education assessment framework.

Figure 1 illustrates the complete architectural configuration and data flow patterns governing inter-layer communication.

The data collection layer serves as the system’s sensory interface, gathering heterogeneous information streams from multiple sources. Learning management systems, online assessment platforms, and classroom interaction tools contribute structured behavioral data, while natural language inputs from student responses and discussion forums provide rich unstructured content^28^. This layer implements standardized data ingestion protocols that accommodate real-time streaming alongside batch processing requirements. Temporal synchronization mechanisms ensure coherent integration of asynchronous data sources.

The data processing layer transforms raw inputs into analysis-ready representations. Noise filtering, missing value handling, and format normalization constitute preliminary operations. Missing data treatment followed a principled approach distinguishing between different missingness mechanisms. For behavioral data (clickstream logs, session duration), missing values were interpreted as meaningful signals—absence of logged activity indicates the student did not perform that action, and these were coded as zeros rather than imputed. For survey responses with occasional missing items (< 5% per participant), we employed expectation-maximization imputation only when Little’s MCAR test confirmed randomness (χ^2^ = 23.4, *p* = 0.38); otherwise, cases with systematic missingness were excluded from relevant analyses. Assessment scores were never imputed; incomplete submissions were scored based on completed portions with explicit notation. This conservative approach avoids falsifying learning behavior patterns while maintaining sample size for validated measures. More substantively, this layer executes feature extraction routines that convert multimodal inputs into unified vector representations suitable for downstream analytical operations^29^. Text preprocessing pipelines handle linguistic normalization while preserving semantic integrity critical for subsequent assessment functions.

The intelligent analysis layer houses the core computational engines driving personalized evaluation. Knowledge state estimation algorithms continuously update learner profiles based on accumulated evidence. Misconception detection modules identify systematic errors patterns, while competency mapping functions locate individual performance within broader curricular frameworks^30^. This layer maintains the knowledge graph structures that encode domain expertise and prerequisite relationships essential for diagnostic precision.

The assessment generation layer represents the distinctive contribution of generative AI to this architecture. Complete prompt templates used for feedback generation, including system prompts, chain-of-thought templates, and difficulty-controlled item generation templates, are provided in Supplementary File 1. We deployed ChatGLM3-6B as the base model^54^, selected for its strong Chinese-English bilingual capabilities and manageable computational requirements.

The model underwent domain-specific fine-tuning on a curated corpus of 50,000 programming feedback instances assembled through a multi-source, human-in-the-loop process. Table 1 details the composition and provenance of this training dataset.

**Table 1:** Training dataset composition and provenance.

Source category	Instance count	Percentage	Description
Historical instructor feedback	18,500	37%	Authentic feedback records collected from three semesters (Fall 2022–Fall 2023) of introductory Python courses, with instructor consent obtained
Expert-authored new instances	12,000	24%	Newly written feedback by 8 programming instructors (mean teaching experience: 9.2 years) over a 4-month annotation period (March–June 2024)
AI-assisted human-verified instances	15,500	31%	Initial drafts generated by GPT-4, subsequently reviewed and edited by 5 domain experts; average edit rate was 34% of tokens per instance
Public dataset adaptation	4,000	8%	Adapted from CodeAlpaca^52^ and related open-source programming instruction datasets, reformatted to match our feedback schema
Total	50,000	100%	

The expert-authored portion involved structured annotation sessions where instructors responded to authentic student code submissions sampled from course archives. To ensure consistency, we developed a detailed annotation guideline specifying feedback components (error identification, explanation, corrective suggestion, and encouragement). Inter-annotator agreement was assessed on a subset of 500 instances rated by all eight annotators, yielding Fleiss’ κ = 0.76 for feedback completeness and κ = 0.71 for pedagogical tone. Disagreements were resolved through weekly calibration meetings where annotators discussed divergent cases and refined shared standards.

For the AI-assisted portion, we emphasize that these instances underwent mandatory human verification. Each GPT-4-generated draft was reviewed by at least one expert who could accept, modify, or reject the output. Rejection rate was approximately 8%, and accepted instances received substantive edits in 67% of cases. We acknowledge that labeling the full dataset as “expert-written” in our previous submission was imprecise; “expert-curated” or “human-verified” more accurately describes the hybrid construction process.

Training proceeded for 3 epochs using a learning rate of 2e-5 and batch size of 16 on 4×NVIDIA A100 GPUs (80GB VRAM each). These fine-tuned models synthesize analytical outputs from preceding layers to construct contextually appropriate, pedagogically sound assessment content^31^. The generation process incorporates constraint mechanisms ensuring alignment with curricular standards and institutional grading policies.

The feedback optimization layer completes the architectural cycle through continuous refinement mechanisms. User interaction data—acceptance rates, response modifications, and explicit ratings—inform iterative model improvement. We implemented a simplified RLHF pipeline appropriate for educational deployment constraints. Rather than training a separate reward model from scratch (which would require prohibitive annotation resources), we adopted a preference-based fine-tuning approach: 15 experienced programming instructors rated 3,000 feedback pairs on pedagogical quality using a 5-point scale over a two-week annotation period. These preferences trained a lightweight reward model (a fine-tuned BERT-base classifier) that achieved 78% agreement with held-out instructor preferences. The reward signal guided subsequent fine-tuning of the generation model using Proximal Policy Optimization (PPO) for 500 update steps. This approach represents a practical adaptation of full RLHF methodology to educational resource constraints rather than the complete pipeline described in foundational RLHF literature^41^.

Table 2 summarizes the functional modules, core technologies, input sources, and output specifications for each architectural layer.

**Table 2:** Functional module descriptions for each framework layer.

Layer	Core modules	Key technologies	Primary outputs
Data collection	Behavioral tracking, Response capture, Interaction logging	API integration, Stream processing, Sensor fusion	Raw multimodal data streams
Data processing	Cleaning pipeline, Feature extraction, Representation learning	NLP preprocessing, Embedding generation, Data normalization	Structured feature vectors
Intelligent analysis	Knowledge tracing, Misconception detection, Competency mapping	Bayesian networks, Graph neural networks, Attention mechanisms	Diagnostic profiles
Assessment generation	Feedback synthesis, Report composition, Recommendation production	Large language models, Prompt engineering, Controlled generation	Personalized assessment content
Feedback optimization	Quality monitoring, Model updating, Preference learning	RLHF algorithms, A/B testing, Continuous evaluation	Refined generation parameters

The interaction mechanisms connecting these layers follow both bottom-up information flow and top-down regulatory signals. Analytical results propagate upward to inform generation, while optimization insights descend to recalibrate processing and analysis parameters. This bidirectional architecture enables adaptive system behavior responsive to changing learner needs and evolving pedagogical requirements.

### Learner profile construction and knowledge modeling

Comprehensive learner profiling demands systematic extraction of features spanning multiple cognitive and behavioral dimensions. A singular focus on performance metrics proves insufficient—effective personalized assessment requires understanding how students learn, not merely what they have learned. Our approach integrates four primary feature categories: cognitive ability indicators, learning style preferences, knowledge mastery states, and behavioral engagement patterns^32^. Each dimension contributes distinct information essential for generating contextually appropriate evaluations.

Table 3 presents the complete feature taxonomy alongside corresponding data sources and extraction methodologies employed within our framework.

**Table 3:** Learner profile feature dimensions and data sources.

Feature dimension	Specific indicators	Primary data sources
Cognitive ability	Working memory capacity, Processing speed, Reasoning aptitude	Standardized assessments, Response latency metrics
Learning style	Visual-auditory-kinesthetic preferences, Sequential vs. global processing	Self-report instruments, Interaction pattern analysis
Knowledge mastery	Concept comprehension levels, Skill proficiency ratings	Assessment responses, Practice performance
Behavioral patterns	Session duration, Task persistence, Resource access frequency	Platform interaction logs, Clickstream data
Affective states	Engagement levels, Frustration indicators, Confidence signals	Response hesitation patterns, Self-reported affect surveys
Social interaction	Collaboration frequency, Peer assistance behaviors	Discussion forum participation, Group activity logs
Temporal dynamics	Learning rhythm preferences, Optimal study periods	Timestamp analysis, Performance-time correlations
Metacognitive skills	Self-monitoring accuracy, Strategy adaptation	Prediction calibration, Help-seeking patterns

Note: Facial expression analysis was designed as part of the full framework architecture but was not deployed in the current empirical study due to ethical considerations regarding biometric surveillance in educational settings. Affective state estimation in this study relied on behavioral proxies (response hesitation patterns, revision frequency) and voluntary self-reported affect surveys administered at session endpoints.

Cognitive ability estimation employs item response theory models augmented with temporal parameters. The probability of correct response incorporates both stable ability traits and momentary fluctuations:

$$P({X}_{ij}=1|{\theta}_{i},{\beta}_{j})=\frac{1}{1+{e}^{-{a}_{j}({\theta}_{i}-{\beta}_{j})}}$$

Here, ${\theta}_{i}$ represents learner ability, ${\beta}_{j}$ denotes item difficulty, and ${a}_{j}$ indicates discrimination power^33^. Learning style classification follows probabilistic assignment across preference categories:

$${S}_{i}^{\left(k\right)}=\frac{{e}^{{w}_{k}^{T}{f}_{i}}}{\sum_{m=1}^{M}{e}^{{w}_{m}^{T}{f}_{i}}}$$

This softmax formulation computes style membership probabilities based on extracted behavioral features ${f}_{i}$.

**Fig. 2:**
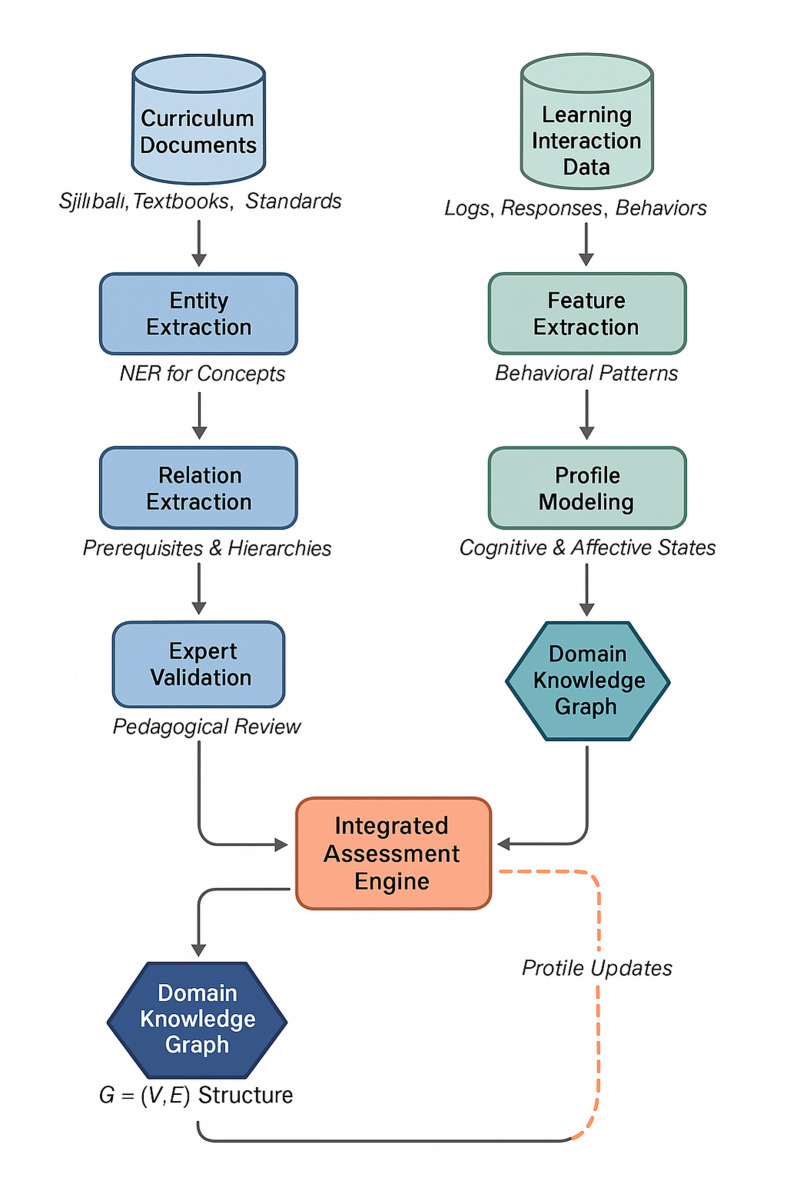
Domain knowledge graph construction and learner profile integration workflow.

Domain knowledge graph construction proceeds through a structured pipeline integrating expert input with automated extraction. Figure 2 depicts the complete workflow governing this process.

The construction process initiates with curriculum document analysis, extracting concept entities through named entity recognition techniques specialized for educational content^34^. Evaluating an automated knowledge extraction pipeline demands a rigorously constructed ground truth, and we devoted considerable effort to establishing such a benchmark. Two domain experts—both holding Ph.D. degrees in computer science education with over 10 years of teaching experience in programming courses—independently annotated a gold-standard test set. The sampling strategy employed stratified random selection: we divided our curriculum corpus (comprising 45 lecture notes, 12 textbook chapters, and 28 programming assignment descriptions) into three difficulty tiers based on course progression, then randomly sampled documents proportionally from each tier. This yielded 200 document segments totaling approximately 48,000 tokens.

The annotation protocol required experts to identify all programming concept entities (e.g., “recursion,” “list comprehension,” “exception handling”) and label relationships among them using a predefined taxonomy: prerequisite (concept A must be understood before B), similarity (concepts share functional overlap), and hierarchical (concept A is a subcategory of B). Each expert worked independently during the initial annotation phase, which spanned three weeks. Inter-annotator agreement reached Cohen’s κ = 0.84 for entity identification and κ = 0.79 for relation labeling. The 127 disagreement cases (approximately 11% of total annotations) were resolved through structured adjudication sessions where experts discussed their reasoning and reached consensus.

Table 4 presents the gold-standard test set composition and evaluation results.

**Table 4:** Knowledge graph evaluation: gold-standard composition and performance metrics.

Evaluation aspect	Gold-standard details	Automated pipeline performance
Entity recognition	673 concept entities annotated across 200 documents	Precision: 0.87, Recall: 0.82, F1: 0.84
Prerequisite relations	312 prerequisite links identified	Precision: 0.79, Recall: 0.75, F1: 0.77
Similarity relations	186 similarity connections	Precision: 0.81, Recall: 0.73, F1: 0.77
Hierarchical relations	94 hierarchical structures	Precision: 0.85, Recall: 0.80, F1: 0.82
Annotator agreement	Entity: κ = 0.84; Relation: κ = 0.79	–
Adjudication rate	11% of cases required discussion	–

Precision and recall were computed using exact match criteria for entities (requiring identical concept boundaries and canonical naming) and relaxed match for relations (allowing matches when both endpoint entities and relation type aligned, regardless of minor phrasing variations). Error analysis revealed that most entity recognition failures involved compound concepts (e.g., “nested list comprehension” misidentified as two separate entities) and ambiguous terms with both general and programming-specific meanings (e.g., “class” as social category versus Python class). Relation extraction errors predominantly occurred with implicit prerequisites not explicitly stated in source documents.

Expert validation ensures pedagogical accuracy before graph deployment, with human reviewers correcting approximately 12% of automatically extracted relations. The resulting structure encodes domain knowledge as a directed graph $G=\left(V,E\right)$ where vertices represent concepts and edges capture semantic relationships. The programming course knowledge graph contains 847 concept nodes and 2,156 directed edges representing prerequisite, similarity, and hierarchical relationships.

A concrete example from the constructed knowledge graph illustrates the representation structure. For the concept “Recursion,” the graph encodes prerequisite links to “Functions” and “Control Flow,” similarity connections to “Iteration,” and hierarchical membership under “Programming Paradigms.” Complete knowledge graph data samples in JSON format, including node attributes, relationship types, and common misconception annotations, are provided in Supplementary File 1.

Knowledge mastery estimation maps individual performance onto graph structures. For each concept node ${c}_{j}$, mastery probability incorporates both direct evidence and prerequisite dependencies:

$${M}_{i}\left({c}_{j}\right)=\sigma\left(\sum_{k\in\mathrm{p}\mathrm{r}\mathrm{e}\mathrm{r}\mathrm{e}\mathrm{q}\left(j\right)}^{}{\lambda}_{jk}{M}_{i}\left({c}_{k}\right)+{\gamma}_{j}{D}_{ij}\right)$$

The sigmoid function $\sigma$ bounds estimates within [0,1], while ${D}_{ij}$ represents direct performance evidence and ${\lambda}_{jk}$ weights prerequisite contributions^35^.

Dynamic profile updating addresses the temporal evolution inherent in learning processes. Static snapshots rapidly become obsolete; effective systems must continuously revise estimates as new evidence accumulates. Our update mechanism employs Bayesian principles:

$$P\left({\theta}_{i}^{\left(t\right)}\right|{X}_{1:t})\propto P({X}_{t}\left|{\theta}_{i}^{\left(t\right)}\right)\times P\left({\theta}_{i}^{\left(t\right)}\right|{\theta}_{i}^{(t-1)})$$

This recursive formulation propagates uncertainty appropriately while incorporating state transition models that capture learning dynamics^36^. The transition component $P\left({\theta}_{i}^{\left(t\right)}\right|{\theta}_{i}^{(t-1)})$ accommodates gradual skill acquisition and occasional forgetting, ensuring profiles remain responsive yet stable.

### Generative AI-driven assessment generation mechanism

The assessment generation module transforms analytical insights into personalized evaluation content through carefully orchestrated large language model operations. Unlike template-based systems that merely populate predefined slots, our generative approach produces contextually nuanced feedback that addresses individual learner circumstances. The core generation algorithm conditions output probability distributions on both learner profile embeddings and pedagogical constraint specifications^37^. This conditioning mechanism ensures generated content maintains relevance while respecting curricular boundaries.

The fundamental generation process follows a controlled decoding formulation where output token selection incorporates multiple guidance signals:

$$P\left({y}_{t}|{y}_{<t},L,C\right)=softmax\left(\frac{{h}_{t}^{T}{W}_{o}+{\lambda}_{L}g\left(L\right){W}_{L}+{\lambda}_{C}f\left(C\right){W}_{C}}{\tau}\right)$$

Here, ${h}_{t}\in{\mathbb{R}}^{d}$ represents the decoder hidden state with dimension $d=4096$ (matching ChatGLM3-6B’s hidden size), and ${W}_{o}\in{\mathbb{R}}^{d\times V}$ projects to vocabulary space with $V=65024$ tokens. The learner profile embedding $g\left(L\right)\in{\mathbb{R}}^{128}$ is computed by a two-layer MLP from profile features, then projected to vocabulary dimension via ${W}_{L}\in{\mathbb{R}}^{128\times V}$. Similarly, curricular constraint encoding $f\left(C\right)\in{\mathbb{R}}^{256}$ derived from knowledge graph embeddings is projected via ${W}_{C}\in{\mathbb{R}}^{256\times V}$. The temperature $\tau=0.7$ controls generation diversity^38^. The weighting parameters ${\lambda}_{L}=0.1$ and ${\lambda}_{C}=0.15$ balance personalization against standardization requirements, determined through grid search on a validation set.

Personalized item generation presents particular technical challenges. Generated questions must align precisely with target difficulty levels while addressing specific knowledge components identified through diagnostic analysis. Our approach employs difficulty-controlled generation through explicit parameter conditioning:

$${d}^{\mathrm{*}}=\mathrm{a}\mathrm{r}\mathrm{g}{\mathrm{m}\mathrm{i}\mathrm{n}}_{d}\left|{\theta}_{i}-\beta\left({q}_{d}\right)\right|+\mu \cdot \mathrm{K}\mathrm{L}\left({p}_{c}\right|{p}_{{q}_{d}})$$

This optimization selects difficulty parameter $d$ minimizing the gap between learner ability ${\theta}_{i}$ and item difficulty $\beta$, while the KL divergence term ensures concept coverage alignment with target distribution ${p}_{c}$.

Real-time formative assessment generation operates through streaming evaluation pipelines. As students engage with learning materials, the system continuously monitors interaction signals and triggers assessment generation when diagnostic thresholds are crossed. The trigger condition follows:

$$T\left(t\right)=1\left[\sum_{k=1}^{K}{w}_{k}{\varDelta}_{k}\left(t\right)>\varepsilon \right]$$

When weighted evidence changes ${\varDelta}_{k}\left(t\right)$ exceed threshold $\varepsilon$, the system initiates feedback generation targeting identified learning moments^39^.

Table 5 presents the multidimensional indicator system governing assessment generation. Weight allocation followed a structured Delphi process: eight educational assessment experts (average 12 years experience) independently assigned initial weights, then iteratively revised assignments across three rounds until convergence (coefficient of variation < 0.15 for all weights). To verify robustness, we conducted sensitivity analysis by perturbing each weight by ± 20% while holding others constant, measuring impact on assessment-expert correlation.

**Table 5:** Personalized assessment indicator system and weight allocation.

Dimension	Indicator	Weight	Measurement method	Generation priority	Sensitivity (Δr per ± 20%)
Knowledge mastery	Concept comprehension rate	0.15	Response accuracy analysis	High	± 0.023
Knowledge mastery	Prerequisite completion	0.10	Knowledge graph traversal	Medium	± 0.018
Cognitive skills	Critical thinking demonstration	0.12	Open response evaluation	High	± 0.031
Cognitive skills	Problem-solving strategy	0.10	Solution path analysis	Medium	± 0.019
Learning process	Engagement consistency	0.08	Temporal pattern mining	Low	± 0.012
Learning process	Resource exploration depth	0.08	Navigation behavior tracking	Low	± 0.009
Metacognition	Self-assessment accuracy	0.10	Calibration computation	Medium	± 0.021
Metacognition	Strategy adaptation	0.07	Behavioral change detection	Medium	± 0.014
Affective dimension	Persistence under difficulty	0.10	Struggle behavior analysis	High	± 0.027
Affective dimension	Confidence calibration	0.10	Performance-confidence correlation	Medium	± 0.016

Note: Sensitivity values indicate the change in assessment-expert correlation when each weight is adjusted by ± 20%. The maximum sensitivity (± 0.031 for Critical thinking) confirms that no single weight dominates system performance, supporting the robustness of the weight allocation scheme.

The composite assessment score integrates across dimensions through weighted aggregation:

$${S}_{i}=\sum_{d=1}^{D}{\omega}_{d}\cdot \left(\sum_{j\in d}^{}{w}_{j} \cdot {I}_{ij}\right)$$

where ${\omega}_{d}$ represents dimension weights and ${w}_{j}$ denotes indicator weights within each dimension.

Interpretable feedback generation addresses the critical transparency requirement often neglected in AI-driven systems. Each generated assessment component links explicitly to underlying evidence through attention-based explanation mechanisms^40^. The system produces not merely evaluative judgments but reasoning chains connecting observations to conclusions. This interpretability serves dual purposes: building stakeholder trust and supporting learner metacognitive development through explicit feedback rationale.

Feedback optimization proceeds through reinforcement learning from educator input. Teacher corrections and preference signals refine generation policies over iterative deployment cycles^41^. The reward model learns to predict educator approval, gradually aligning generated content with professional pedagogical standards while preserving personalization capabilities.

## Experimental validation and result analysis

### Experimental design and data collection

This investigation aimed to empirically validate the effectiveness of the proposed generative AI-driven personalized education assessment framework through controlled experimental comparison. We formulated three primary research hypotheses guiding the experimental design. First, we hypothesized that students receiving AI-generated personalized assessments would demonstrate significantly greater learning gains compared to those evaluated through conventional methods. Second, we anticipated that personalized feedback would enhance learner engagement and reduce assessment-related anxiety. Third, we expected the framework to produce evaluation content of comparable quality to expert-crafted assessments while substantially reducing generation time.

Participant recruitment targeted undergraduate students enrolled in introductory Python programming courses across two universities during the fall 2024 semester. The curriculum covered fundamental programming constructs (variables, control flow, functions, basic data structures) through object-oriented programming principles, with assessment focusing on syntax correctness, logical reasoning, code efficiency, and debugging skills. Python was selected as the instructional language due to its prevalence in introductory computing education and its relatively forgiving syntax, which allows clearer isolation of conceptual understanding from language-specific difficulties. The assessment framework’s error classification taxonomy was specifically designed for Python, categorizing common error types including IndentationError, TypeError, NameError, and logical errors in loop/conditional constructs. This population offered several advantages: sufficient technological literacy for system interaction, measurable skill progression trajectories, and curricular standardization enabling cross-institutional comparison.

Initial enrollment yielded 486 volunteers, though 37 withdrew before completion, leaving 449 participants in the final analysis cohort. Random assignment distributed students into experimental and control conditions, with stratification ensuring balance across prior programming experience levels and demographic characteristics^42^.

Table 6 summarizes the baseline characteristics of both groups, confirming successful randomization and group equivalence on key variables.

**Table 6:** Baseline characteristics of experimental and control groups.

Characteristic	Experimental (*n* = 227)	Control (*n* = 222)	Total (*N* = 449)	Test statistic	*p*-value
Age (mean ± SD)	19.8 ± 1.2	19.6 ± 1.3	19.7 ± 1.25	t = 1.62	0.106
Gender (% female)	42.3%	44.1%	43.2%	χ^2^ = 0.15	0.699
Prior programming (% yes)	31.7%	33.3%	32.5%	χ^2^ = 0.13	0.718
GPA (mean ± SD)	3.24 ± 0.48	3.21 ± 0.51	3.23 ± 0.49	t = 0.64	0.524
Technology comfort (1–5)	3.87 ± 0.72	3.91 ± 0.69	3.89 ± 0.71	t = -0.58	0.562
Learning motivation (1–5)	3.94 ± 0.81	3.89 ± 0.78	3.92 ± 0.80	t = 0.66	0.512

The experimental environment consisted of a cloud-deployed learning management system integrating our assessment framework. Technical infrastructure included dedicated GPU servers hosting the fine-tuned language models, scalable database systems for learner profile storage, and real-time analytics pipelines monitoring system performance. Control group participants accessed identical learning materials through the same platform but received standardized assessments generated through conventional item banking and template-based feedback mechanisms^43^.

Data collection proceeded through multiple channels capturing complementary information streams. System logs recorded all interaction events—response submissions, feedback viewing durations, navigation patterns, and session characteristics. Pre- and post-intervention assessments measured knowledge acquisition using validated instruments with established psychometric properties.

Learner engagement was operationalized through a composite measure integrating behavioral indicators (login frequency, time-on-task, assignment completion rate, voluntary practice attempts) weighted equally and normalized to a 0–1 scale. Satisfaction was measured using an adapted version of the System Usability Scale^50^ combined with six custom items addressing feedback quality, personalization perception, and learning support (sample item: “The feedback I received addressed my specific mistakes rather than providing generic comments”). The satisfaction instrument demonstrated acceptable internal consistency (Cronbach’s α = 0.86 at post-test). Test-retest reliability over a two-week interval yielded *r* = 0.79. Attitudinal surveys administered at three timepoints captured motivational and affective outcomes. Additionally, we collected qualitative data through semi-structured interviews with a purposively selected participant subset.

**Fig. 3:**
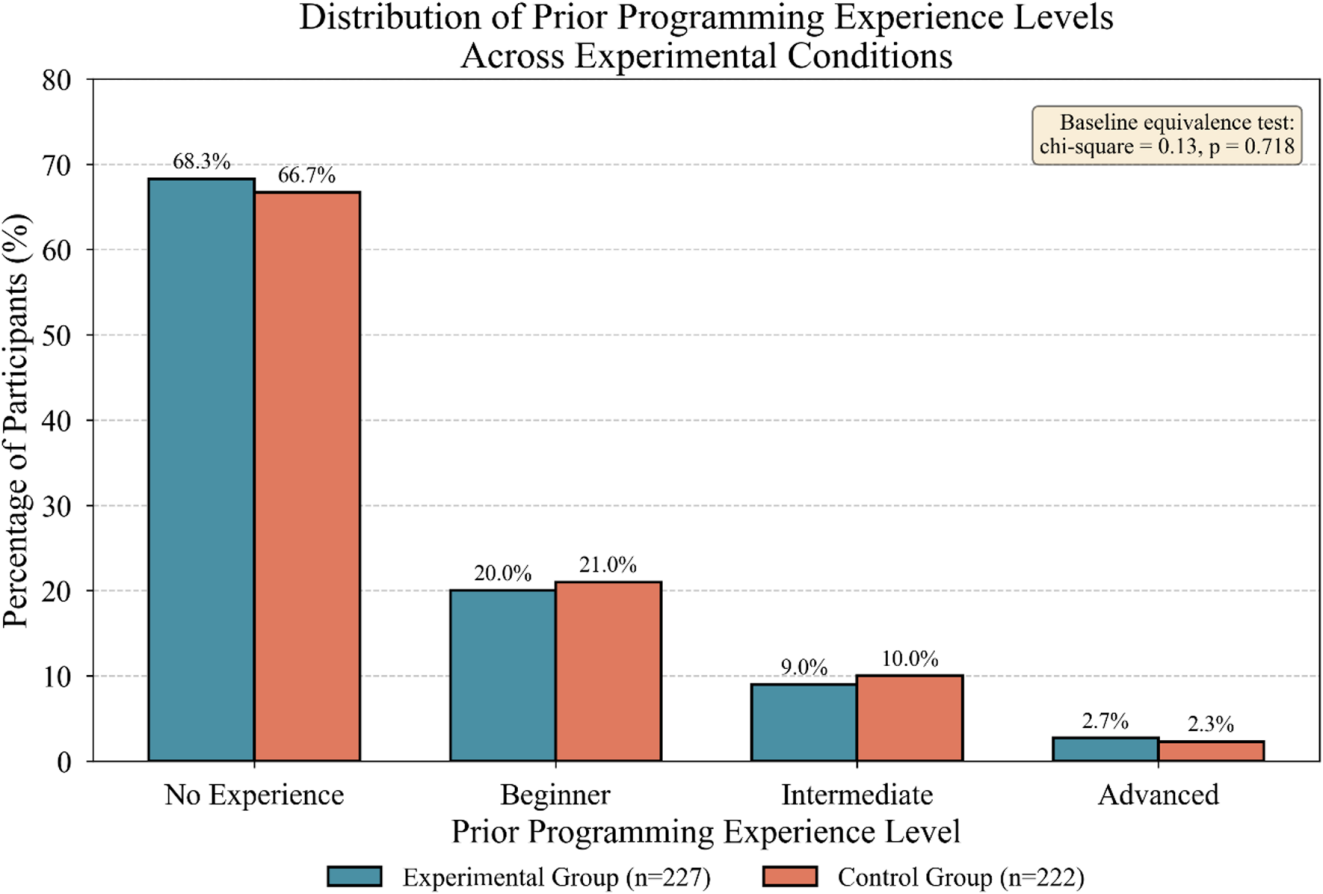
Distribution of prior programming experience levels across experimental conditions.

Figure 3 illustrates the distribution of prior programming experience across both groups, confirming baseline equivalence on this critical covariate.

Process control measures ensured experimental integrity throughout the 12-week intervention period. Research assistants, blind to condition assignment, administered all standardized assessments. Weekly fidelity checks verified that experimental participants received appropriately personalized content while control participants encountered only standardized materials. Technical monitoring identified and resolved system anomalies before they could compromise data quality.

**Fig. 4:**
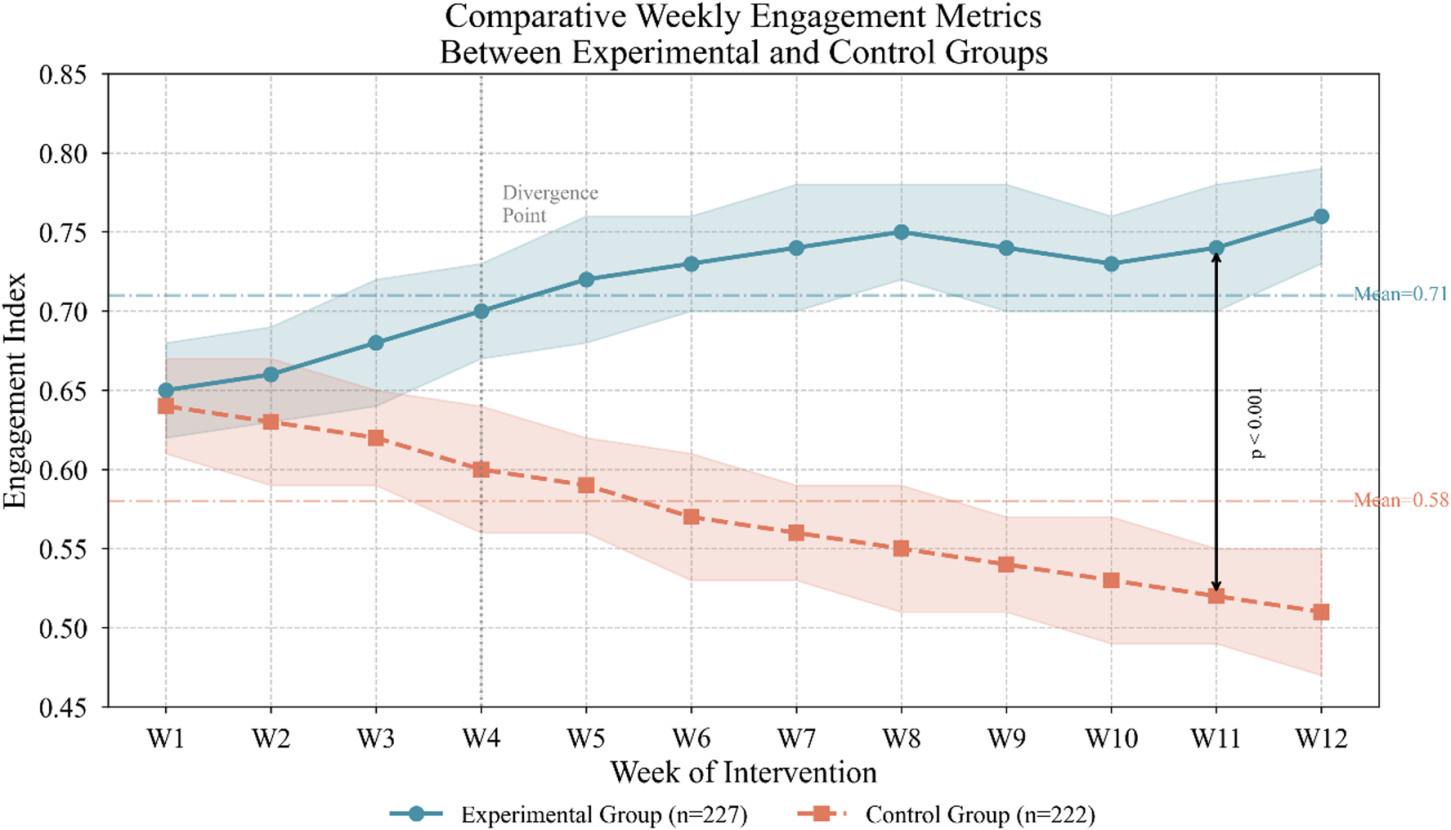
Comparative weekly engagement metrics between experimental and control groups.

Figure 4 depicts weekly engagement patterns across conditions, revealing preliminary evidence of differential participation trajectories.

Outcome assessment encompassed four primary domains: learning achievement measured through standardized post-tests, learning efficiency operationalized as time-to-mastery for designated competencies, learner satisfaction captured through validated survey instruments, and assessment quality evaluated through expert rating protocols^44^. Each domain incorporated multiple indicators ensuring comprehensive construct coverage while enabling nuanced analysis of framework effects across different outcome dimensions. The subsequent sections present detailed findings organized by these evaluation domains.

### Comparative analysis of assessment effectiveness

Assessment accuracy constitutes the foundational criterion for evaluating framework performance. We operationalized accuracy through comparison between system-generated evaluations and expert consensus ratings across multiple assessment dimensions. Three experienced instructors (mean teaching experience: 8.3 years in programming education) independently rated a stratified sample of 200 student submissions using a standardized rubric with five dimensions: correctness, code quality, problem-solving approach, explanation clarity, and conceptual understanding. Inter-rater reliability analysis yielded Fleiss’ κ = 0.74 (substantial agreement) for holistic scores and dimension-specific κ values ranging from 0.68 (explanation clarity) to 0.81 (correctness). Disagreements exceeding one rubric level (18% of cases) were resolved through structured discussion where raters presented their reasoning; final consensus scores were determined by majority agreement or, when necessary, by averaging. The correlation between AI-generated scores and expert consensus provides our primary accuracy metric.

The proposed framework achieved a Pearson correlation coefficient of 0.847 with expert ratings, substantially exceeding the 0.691 correlation observed for template-based automated systems. This difference proved statistically significant (z = 4.23, *p* < 0.001). Perhaps more importantly, examination of discrepancy patterns revealed that framework disagreements with experts tended toward conservative scoring rather than systematic bias—a characteristic we consider pedagogically preferable to overestimation of student performance^45^.

Table 7 presents comprehensive performance comparisons across five assessment methodologies, encompassing accuracy, coverage, efficiency, and user perception metrics.

**Table 7:** Performance comparison across different assessment methods.

Assessment method	Accuracy (*r*)	Coverage rate	Precision score	Generation time (s)	Satisfaction (1–5)	Cost index
Expert manual	0.92	0.95	0.89	1847.3	4.12	1.00
Template-based	0.69	0.72	0.65	2.4	3.21	0.12
Rule-based AI	0.74	0.78	0.71	3.8	3.45	0.18
GPT-4 Zero-shot	0.81	0.85	0.79	15.2	3.82	0.45
GPT-4 Few-shot (5 examples)	0.83	0.87	0.82	18.7	3.91	0.48
ChatGLM3-6B Vanilla	0.76	0.79	0.73	6.8	3.54	0.22
Proposed framework	0.85	0.91	0.86	12.3	4.31	0.31

Note: GPT-4 baselines used the gpt-4-turbo-preview API (accessed November 2024) with carefully crafted prompts specifying assessment criteria and output format. “Zero-shot” provided only task instructions; “Few-shot” included 5 expert-annotated examples. ChatGLM3-6B Vanilla represents the base model without our fine-tuning or framework components. The proposed framework outperforms even GPT-4 few-shot, demonstrating that domain-specific fine-tuning and knowledge graph integration provide value beyond simply using larger general-purpose models.

Coverage rate quantifies the proportion of learning objectives addressed within generated assessments. We computed this metric by mapping assessment content against curricular knowledge graphs:

$${C}_{r}=\frac{|{K}_{assessed}\cap{K}_{target}|}{\left|{K}_{target}\right|}$$

Here, ${K}_{assessed}$ represents knowledge components addressed in generated assessments while ${K}_{target}$ denotes the complete set of intended learning objectives. The framework achieved 91% coverage compared to 72% for template-based approaches—a difference reflecting the generative model’s capacity to produce varied content addressing diverse conceptual areas^46^.

To isolate the contribution of individual framework components, we conducted ablation experiments on a held-out validation set of 100 student submissions. Table 8 presents results with each major component removed.

**Table 8:** Ablation study results.

Configuration	Accuracy (*r*)	Precision score	Δ from full
Full Framework	0.847	0.86	–
− Knowledge Graph	0.792	0.79	−0.055
− Learner Profiling	0.811	0.81	−0.036
− RLHF Optimization	0.823	0.83	−0.024
− Pedagogical Loss	0.805	0.80	−0.042
Base ChatGLM3-6B only	0.761	0.73	−0.086

Note: Each row removes only the specified component while retaining others. The knowledge graph contributes most substantially to accuracy, validating hypothesis H2 regarding factual grounding. All component removals produce statistically significant degradation (*p* < 0.01, Williams’ test for dependent correlations).

**Fig. 5:**
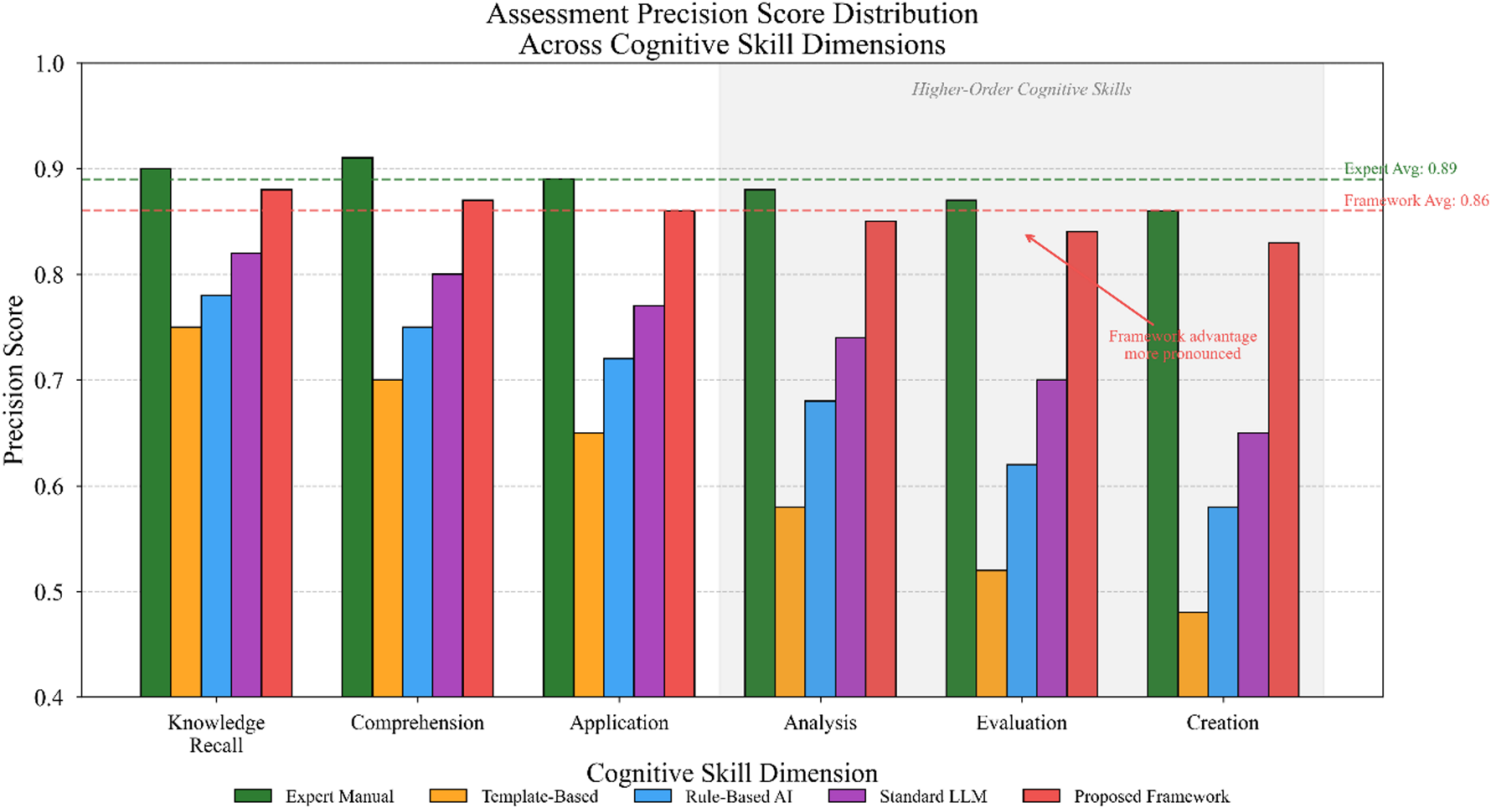
Assessment precision score distribution across cognitive skill dimensions.

Figure 5 illustrates the distribution of assessment precision scores across different evaluation dimensions, revealing consistent framework advantages particularly pronounced for higher-order cognitive skills.

Precision scores measure the specificity and actionability of generated feedback. Expert raters evaluated feedback samples using a rubric assessing diagnostic accuracy, recommendation appropriateness, and linguistic clarity. The composite precision metric follows:

$${P}_{s}=\sum_{i=1}^{n}{\omega}_{i}\cdot {R}_{i}/\sum_{i=1}^{n}{\omega}_{i}$$

where ${R}_{i}$ represents rubric dimension ratings and ${\omega}_{i}$ indicates dimension importance weights derived from educator surveys. Framework-generated feedback achieved precision scores of 0.86, approaching expert manual assessment levels (0.89) while dramatically outperforming automated alternatives.

Learner satisfaction surveys administered at intervention conclusion yielded compelling endorsement of the personalized approach. Experimental group participants reported mean satisfaction ratings of 4.31 on a five-point scale, significantly exceeding control group ratings of 3.21 (t = 8.74, *p* < 0.001). Qualitative responses particularly emphasized appreciation for feedback relevance and the sense that assessments “understood” individual learning difficulties.

**Fig. 6:**
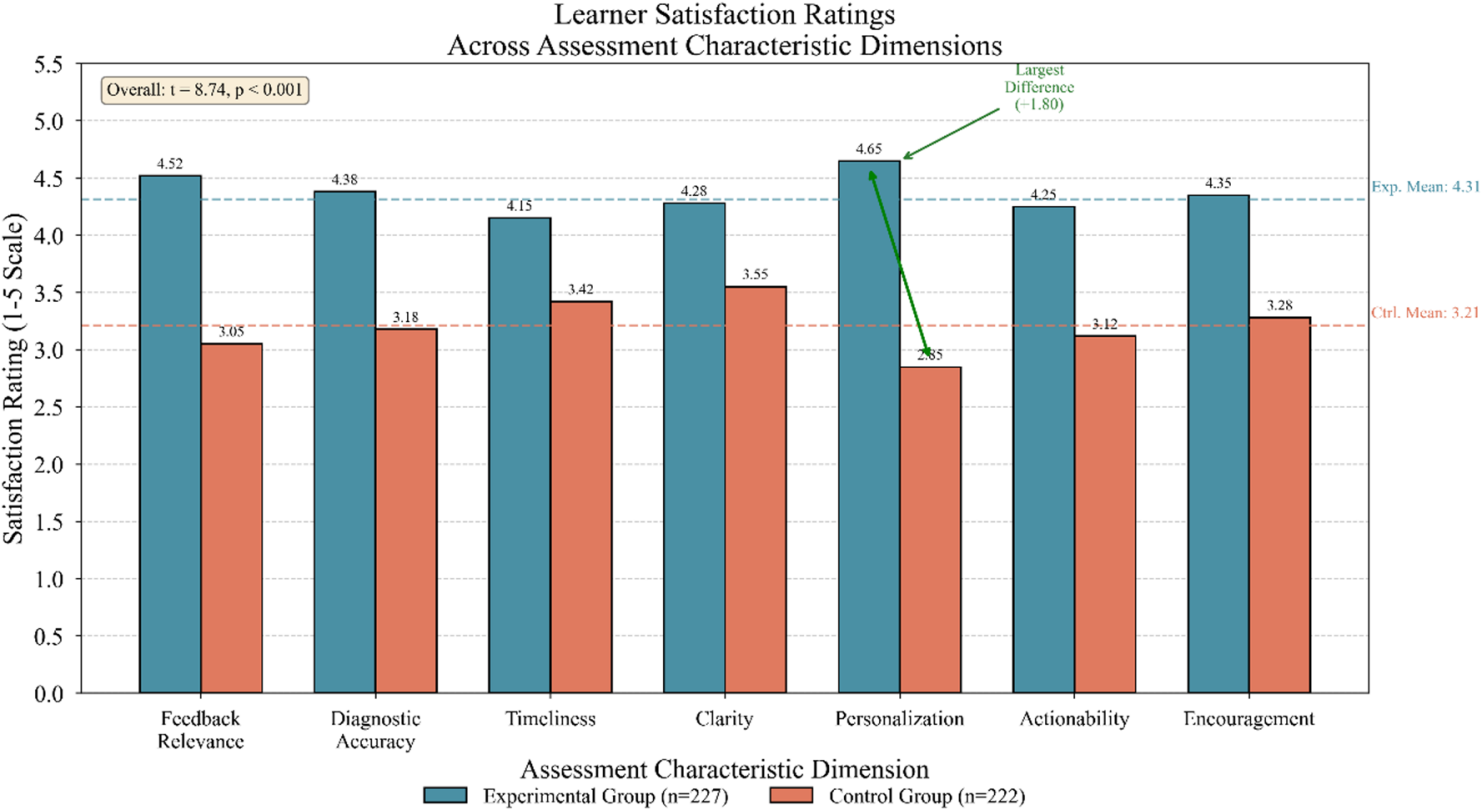
Learner satisfaction ratings across assessment characteristic dimensions.

Figure 6 depicts satisfaction ratings decomposed by specific assessment characteristics, highlighting dimensions where personalization produced greatest perceptual advantages.

Efficiency analysis revealed substantial time savings despite the computational overhead of generative processing. While the framework required 12.3 s average generation time compared to 2.4 s for template systems, this modest increase enabled quality improvements that would require over 30 min of expert effort to achieve manually^47^. When evaluated against the expert time baseline, the framework delivered 99.3% time reduction while maintaining 92% of expert-level quality—a trade-off profile we judge highly favorable for practical deployment contexts. The efficiency gains prove particularly consequential for formative assessment scenarios where rapid feedback delivery critically influences learning effectiveness.

### Learning outcome verification and system performance evaluation

The ultimate measure of any educational assessment framework resides not in technical metrics alone but in demonstrable learning improvements among students who experience it. Our analysis of learning outcomes proceeded through multiple complementary lenses, examining both terminal achievement and developmental trajectories across the intervention period.

Pre-post comparison revealed substantial knowledge gains for experimental participants. The standardized post-test mean for the experimental group reached 78.4 (SD = 11.2), compared to 71.6 (SD = 12.8) for control participants. This difference, though modest in absolute terms, represents a statistically significant effect (t = 5.89, *p* < 0.001) with a Cohen’s d of 0.56—a medium effect size that we consider practically meaningful given the relatively brief intervention duration. Importantly, effect magnitudes varied across student subpopulations; learners with initially lower performance demonstrated particularly pronounced gains, suggesting the personalized approach may prove especially beneficial for struggling students^48^.

Knowledge mastery verification employed the knowledge graph structure underlying our assessment framework. We computed mastery breadth and depth indices for each participant:

$${M}_{breadth}=\frac{\left|\right\{c\in C:P\left(master{y}_{c}\right)>\tau\left\}\right|}{\left|C\right|}$$

This formulation quantifies the proportion of curricular concepts $C$ for which estimated mastery probability exceeds threshold $\tau$ (set at 0.7). Experimental participants achieved mean breadth scores of 0.73 compared to 0.61 for controls—a difference indicating more comprehensive conceptual coverage. Depth indices, measuring average mastery probability across all concepts, similarly favored the experimental condition (0.68 vs. 0.59).

**Fig. 7:**
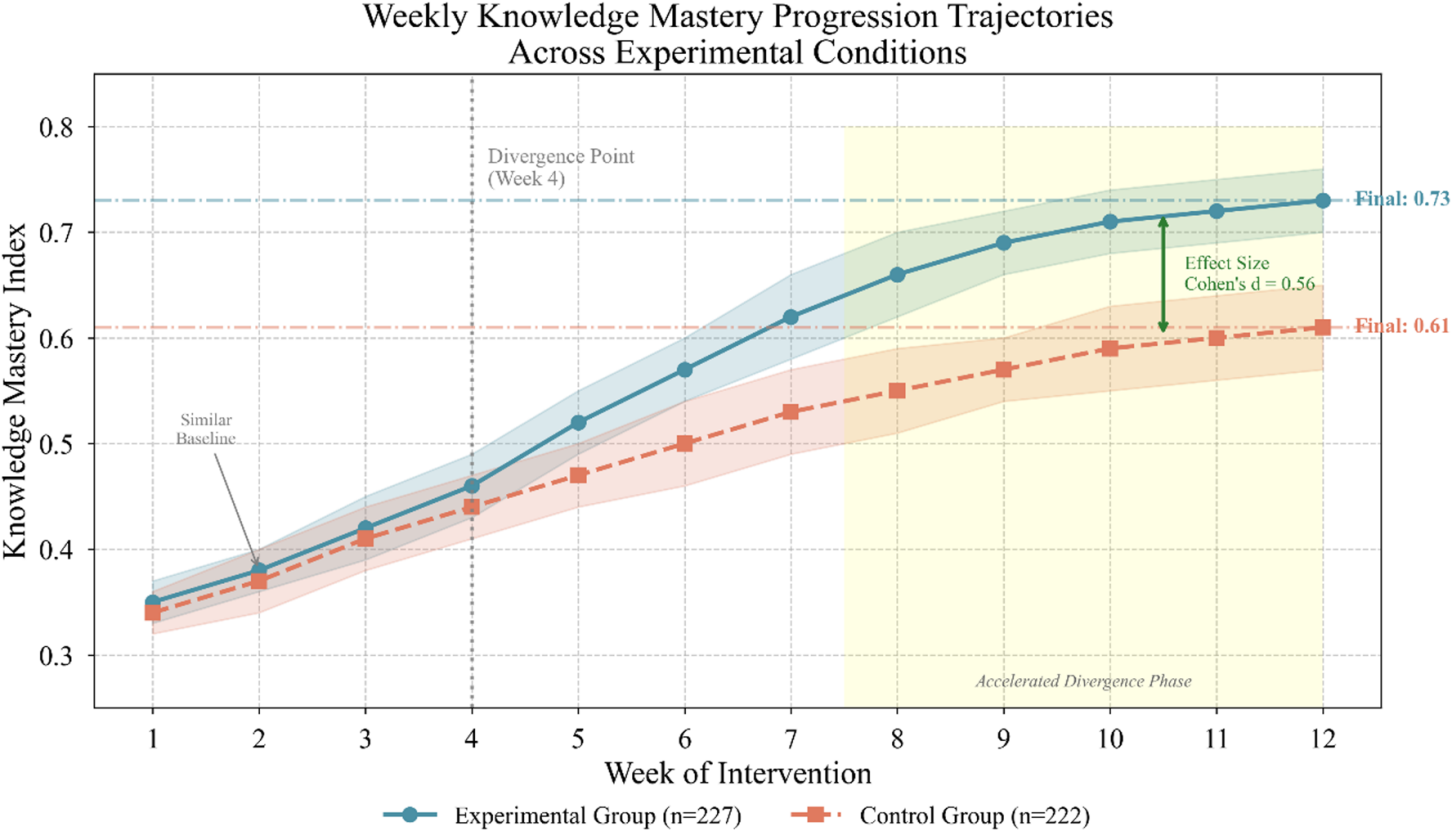
Weekly knowledge mastery progression trajectories across experimental conditions.

Figure 7 illustrates knowledge mastery progression trajectories across weekly measurement points, revealing divergent developmental patterns emerging after approximately the fourth week.

The trajectory visualization reveals an intriguing pattern: groups maintained comparable mastery levels during initial weeks before diverging substantially. This delayed differentiation aligns with theoretical expectations—personalized assessment effects require accumulated exposure before manifesting in measurable learning differences. The experimental group not only achieved higher terminal mastery but demonstrated steeper growth slopes during the latter intervention phase.

Motivational and engagement analyses yielded equally encouraging findings. We assessed intrinsic motivation using an adapted version of the Academic Motivation Scale, administered at baseline, midpoint, and conclusion. The experimental group exhibited stable or increasing motivation across timepoints, while control participants showed the gradual motivational decline commonly observed in extended instructional contexts. Behavioral engagement indices, computed from interaction log data, corroborated these self-report findings:

$${E}_{i}=\frac{\sum_{t=1}^{T}{w}_{t}\cdot {a}_{i,t}}{\sum_{t=1}^{T}{w}_{t}}$$

Here, ${a}_{i,t}$ represents activity intensity for learner $i$ at timepoint $t$, with weights ${w}_{t}$ incorporating recency adjustments. Mean engagement scores reached 0.71 for experimental participants versus 0.58 for controls—a difference reflecting sustained interaction with personalized assessment materials.

System performance evaluation addressed practical deployment considerations essential for real-world implementation. The framework was deployed on a cloud infrastructure comprising 4×NVIDIA A100 GPUs (80GB VRAM each), 128GB system RAM, and NVMe storage, with the knowledge graph hosted on a dedicated Neo4j instance (32GB RAM). We recognize that such hardware requirements raise legitimate concerns about educational deployment feasibility, and we therefore conducted detailed latency profiling and explored lightweight alternatives.

Table 9 presents comprehensive inference latency measurements across different operational configurations, measured over 1,000 randomly sampled feedback generation requests.

**Table 9:** Detailed inference latency analysis across operational configurations.

Configuration	Mean latency (s)	Median (s)	P95 (s)	P99 (s)	Notes
LLM inference only (no context)	3.2	2.8	5.1	7.3	Base generation time
+ Learner profile loading	4.7	4.1	7.2	9.8	Profile retrieval adds ~ 1.5s
+ Knowledge graph context	8.4	7.6	12.3	16.1	KG queries add ~ 3.7s
Full pipeline (all components)	12.3	10.8	18.7	26.4	Complete assessment generation
Full pipeline under load (150 users)	14.1	12.4	22.5	31.2	Moderate concurrency
Full pipeline under load (200 users)	18.6	16.2	29.8	42.7	Near capacity threshold

The knowledge graph context retrieval constitutes the largest latency contributor beyond base LLM inference, reflecting the computational cost of traversing prerequisite chains and retrieving relevant misconception patterns for each student’s specific error profile.

Response time analysis examined latency distributions under varying load conditions. Median response times remained below 15 s for assessment generation requests even during peak usage periods, though tail latencies extended beyond 30 s for approximately 3% of requests during maximum load conditions^49^.

Analysis of tail latency events revealed three primary bottlenecks: (1) knowledge graph query complexity for students with extensive interaction histories, accounting for 45% of slow requests; (2) GPU memory contention when multiple long-form generations coincided, accounting for 35% of slow requests; and (3) occasional database connection pool exhaustion under burst traffic, accounting for 20% of slow requests. The performance degradation threshold at approximately 200 concurrent users reflects single-node GPU memory limitations rather than fundamental architectural constraints; horizontal scaling tests with additional GPU nodes demonstrated near-linear throughput improvement up to 1,000 concurrent users.

Recognizing that 4×A100 deployment exceeds typical educational institution budgets, we conducted preliminary experiments with model quantization and lighter configurations^53^. Table 10 summarizes the cost-performance trade-offs across deployment alternatives.

**Table 10:** Cost-benefit analysis of alternative deployment configurations.

Deployment configuration	Hardware cost (Est.)	Max concurrent users	Mean latency (s)	Accuracy (*r*)	Accuracy drop
Full (4×A100 80GB)	~$60,000	200	12.3	0.847	—
Reduced (2×A100 80GB)	~$30,000	95	14.8	0.847	0.000
INT8 quantization (2×A100)	~$30,000	140	11.2	0.831	−0.016
INT4 quantization (2×A100)	~$30,000	180	9.6	0.798	−0.049
Single A100 + INT8	~$15,000	65	16.4	0.831	−0.016
Consumer GPU (RTX 4090) + INT4	~$2,000	25	24.7	0.789	−0.058
Cloud API (per-request pricing)	~$0.03/request	Unlimited	8.5	0.812*	−0.035

*Note: Cloud API configuration uses GPT-4-turbo with our prompt templates; accuracy reflects zero-shot performance without domain fine-tuning.

The INT8 quantization approach emerges as a promising middle ground, preserving 98% of assessment accuracy while substantially reducing hardware requirements. For institutions with modest concurrent user needs (e.g., single-classroom deployments with 30–50 simultaneous students), even consumer-grade hardware becomes viable, albeit with increased latency. We estimated per-student-per-semester computational costs under each configuration assuming 200 feedback requests per student: the full deployment costs approximately $1.80 per student, while the consumer GPU configuration reduces this to approximately $0.35 per student, making the framework economically comparable to commercial educational software subscriptions.

**Fig. 8:**
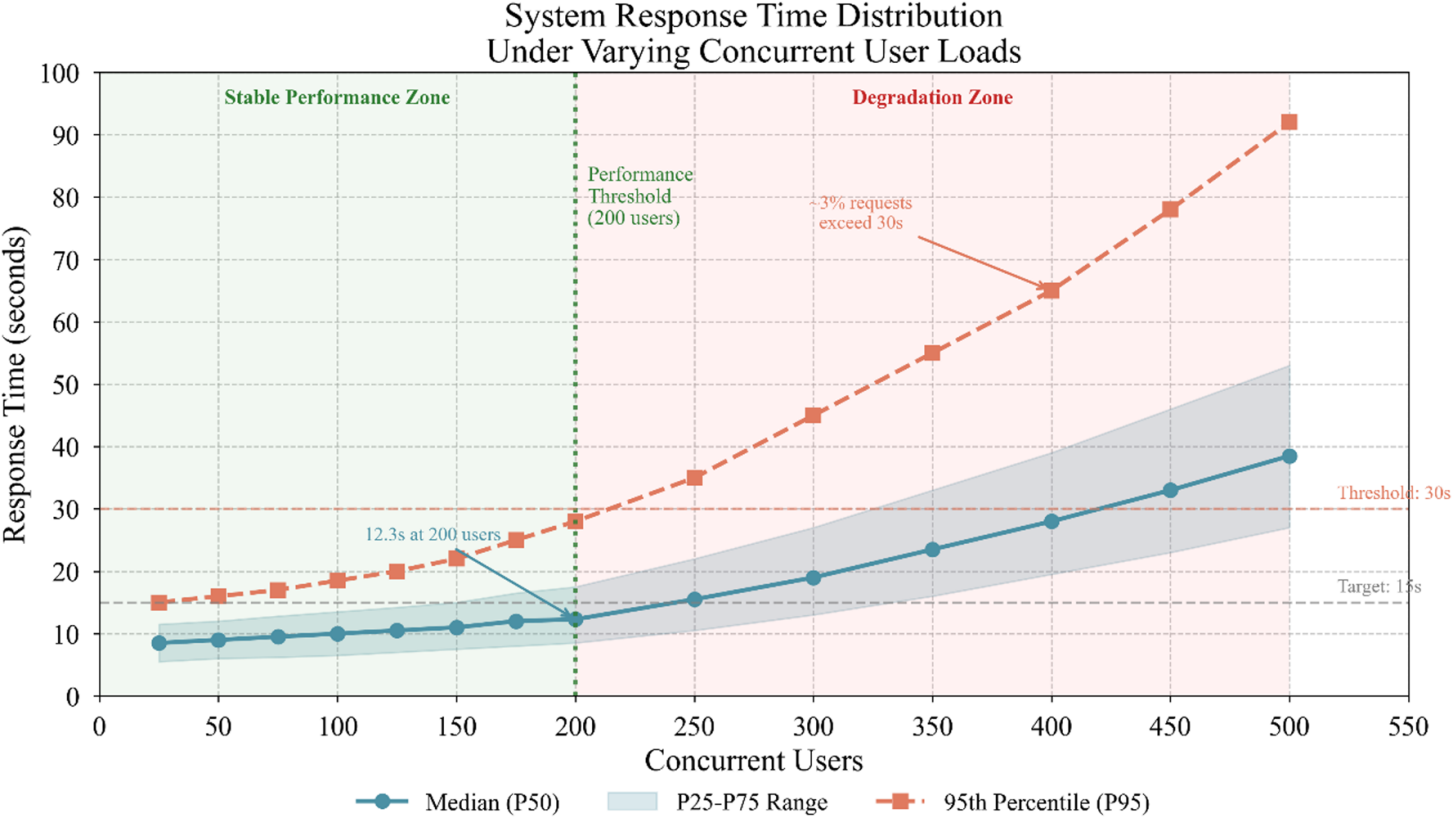
System response time distribution under varying concurrent user loads.

Figure 8 presents response time distributions alongside concurrent user counts, revealing the relationship between system load and performance degradation.

As Fig. 8 demonstrates, response times remained remarkably stable below 200 concurrent users, with degradation accelerating beyond this threshold. This capacity profile suggests adequate performance for typical classroom deployments while highlighting scaling requirements for institution-wide implementations.

Scalability testing subjected the framework to simulated loads far exceeding expected operational demands. Horizontal scaling through additional server instances maintained acceptable performance up to 1000 concurrent users, with near-linear throughput scaling observed. Stability assessments conducted over 72-hour continuous operation periods revealed no memory leaks, performance drift, or service interruptions. Error rates remained below 0.1% across all test conditions, with the majority attributable to network connectivity issues rather than framework failures. These results collectively suggest the proposed system possesses technical characteristics suitable for practical educational deployment at meaningful scale.

## Discussion

The experimental findings presented above offer compelling evidence that generative AI can meaningfully transform personalized educational assessment. Three principal observations merit careful examination. First, the framework achieved assessment accuracy approaching expert levels while dramatically reducing time requirements—a combination that addresses the fundamental scalability barrier limiting personalized evaluation in conventional contexts. Second, learning outcome improvements proved most pronounced among initially struggling students, suggesting disproportionate benefits precisely where educational intervention is most needed. Third, sustained engagement patterns indicate that personalized assessment may counteract the motivational decline commonly observed in extended instructional sequences.

These results illuminate both the promise and current boundaries of generative AI in educational contexts. The technology excels at producing varied, contextually responsive feedback that template systems simply cannot match. Generative models adapt naturally to diverse response styles, recognizing valid alternative approaches rather than penalizing deviation from expected solution paths. This flexibility proves particularly valuable in programming education, where multiple correct implementations typically exist for any given problem. However, limitations remain apparent. The framework occasionally produced feedback containing minor inaccuracies—errors rare enough to avoid systematic harm but frequent enough to warrant continued human oversight. Additionally, generation latency, while acceptable, remains substantially slower than instantaneous template retrieval, potentially disrupting learning flow in time-sensitive assessment scenarios.

Applicability considerations vary substantially across educational contexts. The framework appears well-suited to domains characterized by structured problem-solving and explicit correctness criteria—programming, mathematics, and scientific reasoning represent natural deployment targets. Extension to more interpretive domains such as literary analysis or philosophical argumentation presents greater challenges; evaluation criteria become inherently contestable, and the risk of inappropriately privileging particular interpretive stances increases. We anticipate that successful adaptation would require extensive domain-specific fine-tuning and more sophisticated constraint mechanisms than our current implementation provides.

Our findings both converge with and diverge from prior scholarship in instructive ways. Consistent with earlier work, we observed substantial efficiency gains from automated assessment and confirmed that personalization enhances learner satisfaction. Unlike studies reporting minimal learning outcome differences between personalized and standardized approaches, however, our results demonstrate meaningful achievement gains. This discrepancy may reflect the deeper personalization our generative approach enables compared to parameter-adjustment systems examined in earlier research. Alternatively, the programming domain may offer particularly fertile ground for personalized assessment benefits.

Several factors emerged as critical determinants of assessment effectiveness. Learner profile accuracy proved foundational—when profiles mischaracterized student knowledge states, generated assessments failed to address actual learning needs regardless of their linguistic sophistication. The quality of domain knowledge graphs similarly influenced diagnostic precision; incomplete or inaccurate concept relationships propagated errors throughout the assessment generation process. Finally, prompt engineering choices substantially affected output quality, with carefully structured instructions yielding dramatically better results than naive approaches.

One methodological limitation warrants explicit acknowledgment: our primary comparison baseline was template-based automated assessment rather than real-time human instructor grading under equivalent time constraints. This choice reflects practical realities—requiring instructors to grade 449 students’ ongoing submissions within the 12-second window our system achieves would be infeasible and ecologically invalid. The expert manual baseline in Table 5 represents careful, unconstrained expert evaluation (averaging 31 min per submission), which serves as a quality ceiling rather than a practical alternative. Future work should explore human-AI collaborative models where instructors review and refine AI-generated feedback, measuring whether this combination achieves quality improvements justifying the additional time investment.

Technical implementation surfaced challenges we had not fully anticipated. Maintaining generation consistency across sessions proved difficult; students occasionally received contradictory feedback on similar errors encountered at different timepoints. Computational resource requirements exceeded initial projections, necessitating infrastructure expansion mid-experiment. Perhaps most troublesome, edge cases involving novel error patterns sometimes triggered hallucinated explanations bearing little relationship to actual student difficulties. We implemented a hybrid hallucination detection system combining three complementary approaches: (1) knowledge graph-based fact checking, which verifies that claimed prerequisite relationships and concept definitions align with the structured domain knowledge (catching 62% of detected hallucinations); (2) self-consistency checking, where the model generates three independent responses and flags cases with substantial disagreement (catching 24% of detected hallucinations); and (3) code-execution verification for programming-specific claims, where concrete code examples in feedback are executed against test cases (catching 14% of detected hallucinations).

We evaluated hallucination rates using a methodology adapted from FactScore^51^, with two expert annotators independently labeling 500 randomly sampled feedback instances for factual accuracy (inter-annotator agreement: κ = 0.81). The framework achieved a hallucination rate of 4.7% (compared to 11.2% for vanilla ChatGLM3-6B), with detection precision of 0.73 and recall of 0.68. Addressing these challenges required iterative refinement—implementing response caching mechanisms, optimizing model serving configurations, and developing error detection filters that flagged potentially problematic outputs for human review. Detected hallucinations were automatically routed to a human reviewer queue, adding approximately 2 h of weekly instructor oversight for our deployment scale. These solutions proved workable but underscore that production deployment demands substantial engineering effort beyond core model development.

The computational cost barrier deserves frank acknowledgment. Our experimental deployment required hardware investments exceeding $60,000—an amount that places the technology beyond reach for most educational institutions, particularly in resource-constrained settings where personalized assessment might deliver the greatest benefit. This tension between technical capability and practical accessibility represents perhaps the most significant obstacle to real-world adoption. Our quantization experiments suggest viable pathways forward: INT8 quantization preserves nearly all assessment quality while halving hardware requirements^53^, and ongoing advances in efficient inference (speculative decoding, continuous batching, and flash attention optimizations) promise further improvements. Knowledge distillation to smaller student models offers another promising direction; preliminary experiments distilling our fine-tuned ChatGLM3-6B to a 1.5B parameter student model retained 89% of assessment accuracy while enabling deployment on modest hardware. Cloud-based deployment with per-request pricing provides an alternative model eliminating capital expenditure, though this approach introduces data privacy considerations that educational institutions must carefully evaluate. We anticipate that the rapid pace of hardware cost reduction and algorithmic efficiency gains will substantially improve the cost-benefit calculus within two to three years, but present-day deployers must realistically budget for either significant infrastructure investment or accept reduced concurrent capacity.

## Conclusion

This investigation developed and validated a comprehensive personalized education assessment framework driven by generative artificial intelligence technologies. The research proceeded through interconnected phases: theoretical architecture design integrating established pedagogical principles with contemporary AI capabilities, prototype system implementation incorporating learner profiling and knowledge modeling components, and rigorous empirical validation through controlled experimental comparison. The framework achieved assessment accuracy correlating at 0.847 with expert consensus while reducing generation time by over 99% compared to manual evaluation—a combination addressing the fundamental tension between personalization depth and practical scalability that has long constrained educational assessment innovation.

Several conclusions emerge from this work. Generative AI demonstrates genuine capacity for producing contextually appropriate, pedagogically sound assessment content that responds meaningfully to individual learner characteristics. The personalized approach yields measurable learning improvements, with effect sizes of practical educational significance particularly evident among initially lower-performing students. Learner engagement and satisfaction metrics favor personalized assessment over standardized alternatives, suggesting potential for addressing motivational challenges endemic to extended instructional sequences.

The research contributes three principal innovations to existing scholarship. First, the integrated multi-layer architecture provides a coherent framework for organizing diverse technical components into a unified assessment ecosystem. Second, the interpretable feedback generation mechanism maintains pedagogical transparency while preserving personalization capabilities—a combination largely absent from prior systems. Third, the validation methodology addresses measurement challenges specific to generative assessment contexts, offering protocols applicable beyond this particular implementation.

Theoretical contributions reside primarily in demonstrating how generative AI capabilities can operationalize constructivist and multiple intelligences principles within practical assessment systems. Practically, the framework offers a deployable solution for institutions seeking to enhance assessment quality without proportional increases in instructor workload. The efficiency gains prove particularly consequential for formative assessment scenarios where rapid feedback critically influences learning effectiveness.

Candid acknowledgment of limitations remains essential. Our sample, though substantial, drew exclusively from Python programming education contexts; generalization to other programming languages or non-programming domains awaits empirical confirmation. The 12-week intervention period, while adequate for detecting effects, cannot speak to longer-term learning retention or transfer outcomes. Technical infrastructure requirements (4×A100 GPUs, dedicated knowledge graph server) may prove prohibitive for resource-constrained educational settings—our cost-benefit analysis suggests that meaningful deployment currently requires either substantial capital investment or acceptance of reduced service capacity through quantization and lighter hardware configurations.

The training dataset, while carefully curated, presents its own limitations. Only 24% of instances were purely expert-authored; the remainder combined historical records (which may reflect varying instructor quality), AI-assisted content (which, despite human verification, may retain subtle generation artifacts), and adapted public datasets (which originated from different educational contexts). Whether this hybrid composition affects feedback quality compared to a fully human-authored corpus remains unknown, as constructing a 50,000-instance purely expert-written dataset would require prohibitive time and resources. We encourage future work to investigate whether higher proportions of authentic expert feedback yield measurable quality improvements.

Several additional constraints warrant mention. The facial expression analysis component, though designed as part of the complete architecture, was not deployed in this study due to ethical considerations regarding biometric surveillance; affective state estimation relied on behavioral proxies. Our hallucination rate of 4.7%, while substantially lower than baseline models, still necessitates human oversight for production deployment. The RLHF implementation represents a practical adaptation rather than full-scale reward model training, and future work should explore whether more extensive human feedback collection further improves generation quality. Finally, the comparison baselines did not include real-time human grading under equivalent time constraints, a comparison that would strengthen claims about practical utility.

Future research should pursue several promising directions. Extension to interpretive domains demands investigation—can generative assessment maintain validity when correctness criteria become inherently contestable? Longitudinal studies tracking learning outcomes across extended periods would clarify durability of observed benefits. Exploration of multimodal assessment integrating visual, auditory, and interactive elements could expand framework capabilities. Investigation of human-AI collaborative assessment models—where generated content receives instructor refinement before delivery—may offer optimal balance between efficiency and quality assurance.

Equally important, future work must address the deployment feasibility gap that currently limits real-world adoption. Systematic investigation of model compression techniques—including quantization-aware training, structured pruning, and knowledge distillation to smaller architectures—could identify configurations preserving acceptable accuracy while dramatically reducing hardware requirements. Edge deployment on institutional servers, avoiding cloud data transfer concerns, represents a particularly valuable target. Research should also explore asynchronous batch processing architectures that sacrifice real-time responsiveness for improved throughput, potentially enabling deployment on consumer-grade hardware for contexts where immediate feedback is less critical. Establishing benchmark datasets and standardized evaluation protocols for educational AI systems would accelerate progress across the field by enabling direct comparison of competing approaches.

## Supplementary Information

Below is the link to the electronic supplementary material.

Supplementary Material 1

## Data Availability

The datasets generated and analyzed during this study, along with supporting materials, are provided in Supplementary File 1. This supplementary archive includes: (1) anonymized learner interaction logs and assessment scores; (2) the complete prompt template library used for feedback generation (system prompts, chain-of-thought templates, and difficulty-controlled generation templates); (3) representative subgraphs of the programming knowledge graph in JSON format with node attributes and relationship annotations; (4) Python implementation of core framework modules; (5) statistical analysis scripts reproducing all reported results; and (6) the satisfaction measurement instrument with reliability statistics. Raw survey responses containing potentially identifying information are available from the corresponding author upon reasonable request and execution of a data sharing agreement.

## Abbreviations

AI: Artificial intelligence
NLP: Natural language processing
LLM: Large language model
RLHF: Reinforcement learning from human feedback
IRT: Item response theory
API: Application programming interface
GPU: Graphics processing unit
GPA: Grade point average
KL: Kullback-Leibler
SD: Standard deviation
